# Old case, new leads: miRNA links Kaposi's sarcoma-associated herpesvirus with sepsis

**DOI:** 10.1038/cddis.2014.516

**Published:** 2014-12-04

**Authors:** W-T Lu, M Bushell

**Affiliations:** 1 MRC Toxicology Unit, Hodgkin Building, Leicester, UK

Sepsis is an age-old medical problem. Having first been described by the ancient Greeks, it still troubles us today as one of the most common causes of death in hospitalized patients, with a mortality rate between 20 and 40%.^1^ Sepsis is an umbrella term defining a systemic inflammatory response to presumed or proven infection.^2^ Depending on the severity of complications, sepsis can be further defined as severe sepsis (complicated by remote organ dysfunction) or septic shock (complicated by arterial hypotension).^3^ The initial clinical diagnosis of sepsis is largely dependent on clinical history and bedside examination, supplemented by diagnostic biomarkers such as blood C-response proteins, lactate levels, other inflammatory markers, and ultimately demonstration of infective cause by microbiological blood or other tissue cultures.^1, 3^ These key microbiological tests in particular take some days to complete. Certain patient groups are at particular risk of sepsis, such as those with chronic conditions, such as cancer or obesity, or patients undergoing immune-suppressive treatments are more susceptible to sepsis.^2^ Often, however, young healthy patients are also affected by severe sepsis, and it would seem that there are unknown genetic or environmental factors. Therefore, establishing novel rapid biomarkers of sepsis would potentially be of great benefit to identify early stages for clinical intervention, or to help high-risk populations that are susceptible to severe sepsis or septic shock.

MicroRNAs (miRNAs) are a class of small noncoding RNAs that are between 21 and 25 nucleotides in length. The primary transcripts of miRNAs (pri-miRNAs) are transcribed by RNA polymerase II, and processed into hairpins by Drosha-DGCR8 the microprocessor, then exported to the cytoplasm by Exportin5.^4^ Once in the cytoplasm, they are recognised by Dicer, which cleaves the hairpin structures into double-stranded RNAs (dsRNAs). The dsRNA will then be loaded onto one of the Argonaute (Ago) family of proteins, which unwinds the dsRNA structures and facilitate pairing between miRNA to mRNA. Once the miRNA is in complex with Ago proteins, they bind to target sequences that are mainly located within the 3′ untranslated region (UTR) of a target mRNA.^4, 5^ Following target recognition, miRNA in complex with the Ago protein promotes recruitment of TNRC6 family proteins and the Ccr4-NOT complex, which in turn serve as docking sites for effector proteins.^5^ Recently, data from several groups showed that this recruitment prevents the message from being translated, by inhibiting eIF4F complex scanning the 5′ UTR.^6, 7^ Following that, the mRNA is then decapped, deadenylated and degraded.^6, 7^ Owing to the importance and versatility of the miRNA pathway, some viruses exploit this mechanism to benefit their propagation. One example is Hepatitis C virus (HCV) that hijacks miR-122 to facilitate the replication of its viral RNA.^8, 9^ Yet another example is KSHV that produces 25 viral miRNAs. Among these miRNAs, miR-K12-11 can mimic host miR-155 resulting in aberrant regulation of some key proteins in inflammatory response pathways.^9^

In the current issue of *Cell Death and Disease*, Tudor *et al.*^11^ look into the utility of miRNAs as diagnostic biomarkers in sepsis. They screened for potential differences of miRNA levels in the monocytes of eight healthy and sepsis patients using miRNA microarray. After statistical analysis, a list of 10 cellular miRNAs and 2 viral miRNA were selected for clinical studies. Among these miRNAs, they observed a downregulation of miR-146 and miR-150 plasma levels in sepsis patients. However, this was inconsistent between their cohorts.^10^ The authors claim this may be attributed to differences in sepsis etiology between cohorts. However, especially in the light of conflicting results shown in a previous European study,^11^ larger scale population studies should be carried out to confirm these findings, only then can one hope to use these human miRNAs as potential biomarkers for sepsis.

Surprisingly, Tudor *et al.*^10^ also identify two viral miRNAs as potential biomarker of sepsis, KSHV-miR-K12-10b and KSHV-miR-K12-12*. Within both cohorts, it was observed that KSHV-miR12-10b detection correlates well with the severity of sepsis. In addition to this, they identified significant increases in interleukin (IL)-6 and IL-10 plasma levels from sepsis patients.^10^ Furthermore, this increase of plasma IL-6/10 level also correlates well with the severity of sepsis in patients.^10^ This agrees with other reports claiming that plasma levels of cytokines, especially IL-6, may serve as good prognostics markers to predict the onset and outcome of sepsis.^12, 13^ To evaluate the relationship between KSHV-miR-K12-10b, KSHV-miR-K12-12* and cytokine production, the authors overexpress these two KSHV miRNAs in HEK293 cells. Crucially, upon overexpression of either miRNA, they observed an increase in secretion level of IL-6 and IL-10, at a level that is comparable to lipopolysaccharide (LPS) stimulation.^10^ Given that nearly half of sepsis cases are triggered by Gram-negative bacterial infections and LPS is the major component of its outer membrane,^14^ this finding suggests KSHV infection may be a potential cause of sepsis.

How do these KSHV miRNAs contribute to the pathogenesis of sepsis? The authors suggest that they may not function through the canonical mechanism via post-transcriptional repression of mRNA. Rather, they propose that these miRNAs may serve as ligands for TLR8 and trigger a proinflammatory response, similar to previous reports.^15^ To this end, they carried out RNA-immunoprecipitation (RIP) experiment with Flag-tagged TLR8 and observed an increased binding of KSHV-miR-K12-10b and KSHV-miR-K12-12* to TLR8 compared with other miRNAs.^10^ However, they did not carry out Ago2 RIP experiment, nor investigate potential targets of these two miRNAs. Thus, the possibility that these miRNAs may modulate the host immune response by post-transcriptional regulation should not be dismissed (see Figure 1 for a summary diagram).

As perioperative infection is a common cause of sepsis, the authors questioned whether the increase of KSHV miRNA in sepsis patients is correlated with surgery. Interestingly, using a cohort consisting of nonsepsis patients, the authors observed a significant increase of both KSHV-miR-K12-10b and KSHV-miR-K12-12* plasma level in 71% of patients at post-operative day 1.^10^ This spike of KSHV miRNA dissipated 7 days after operation.^10^ Moreover, they observed a trend showing that the increase of plasma KSHV miRNA level is greater in traditional open abdominal surgery compared with minimally invasive surgery.^10^ The authors suggest that surgical trauma may trigger reactivation of latent KSHV in patients, resulting in a subsequent increase of KSHV miRNA. It is known that KSHV prevalence is variable, with serological positivity for KSH of up to 50% among blood donors in Uganda as compared with 6% in the US.^16, 17^ By any standard, the 71% KSHV miRNA positive rate at post-operative day 1 is very surprising. This needs to be further confirmed using larger patient cohorts and correlated with other tests for KSHV infection, such as seropositivity. Is it possible that surgical trauma is reactivating latent subclinical virus? It is known that KSHV can contribute to malignancy, especially Kaposi's sarcoma. However, malignancy is a rare event after KSHV infection. Therefore, the current finding by Tudor *et al.* that potentially links KSHV to sepsis, warrants further investigation.

## Figures and Tables

**Figure 1:**
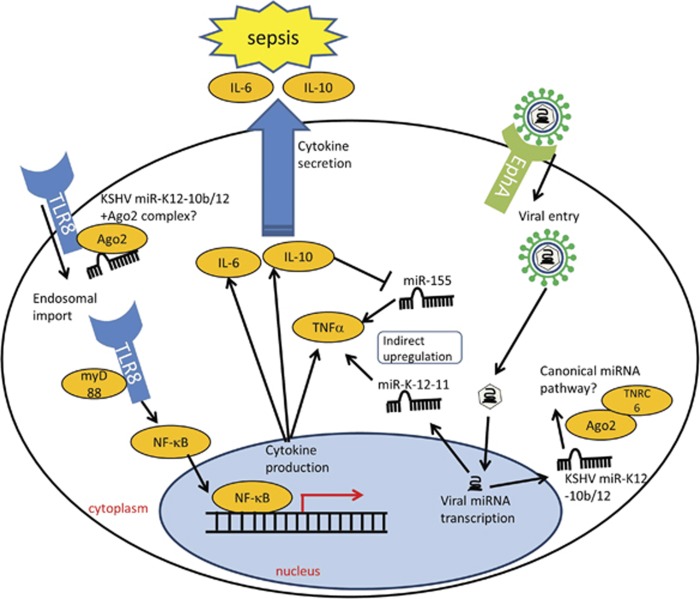
KSHV binds the EphrinA (EphA) receptor.^18^ Viral binding leads to viral internalization via endocytosis, resulting in the viral genome being released into the nucleus.^18^ KSHV produces 25 viral miRNAs.^9^ Tudor *et al.*^10^ suggest that miR-K12-10b/12 may bind to TLR8 and activate cytokine production, in particular IL-6 and IL-10. It is unknown whether these two viral miRNAs post-transcriptionally regulate any host mRNA. Nevertheless, KSHV also transcribes miR-K12-11, which shares the same sequence as miR-155.^9^ This may induce the upregulation of TNF*α* proteins and further activation of the proinflammatory response, triggering sepsis
